# YOLO Algorithm for Long-Term Tracking and Detection of Escherichia Coli at Different Depths of Microchannels Based on Microsphere Positioning Assistance

**DOI:** 10.3390/s22197454

**Published:** 2022-09-30

**Authors:** Lesheng Sun, Ying Xu, Zhikang Rao, Juntao Chen, Zhe Liu, Ning Lu

**Affiliations:** School of Automation, Hangzhou Dianzi University, Hangzhou 310018, China

**Keywords:** 3D relocation, identification of bacteria, deep learning, microfluidics, detection of antibiotic susceptibility

## Abstract

The effect evaluation of the antibiotic susceptibility test based on bacterial solution is of great significance for clinical diagnosis and prevention of antibiotic abuse. Applying a microfluidic chip as the detection platform, the detection method of using microscopic images to observe bacteria under antibiotic can greatly speed up the detection time, which is more suitable for high-throughput detection. However, due to the influence of the depth of the microchannel, there are multiple layers of bacteria under the focal depth of the microscope, which greatly affects the counting and recognition accuracy and increases the difficulty of relocation of the target bacteria, as well as extracting the characteristics of bacterial liquid changes under the action of antibiotics. After the focal depth of the target bacteria is determined, although the z-axis can be controlled with the help of a three-dimensional micro-operator, the equipment is difficult to operate and the long-term changes of the target bacteria cannot be tracked quickly and accurately. In this paper, the YOLOv5 algorithm is adopted to accurately identify bacteria with different focusing states of multi-layer bacteria at the z-axis with any focal depth. In the meantime, a certain amount of microspheres were mixed into bacteria to assist in locating bacteria, which was convenient for tracking the growth state of bacteria over a long period, and the recognition rates of both bacteria and microspheres were high. The recognition accuracy and counting accuracy of bacteria are 0.734 and 0.714, and the two recognition rates of microspheres are 0.910 and 0.927, respectively, which are much higher than the counting accuracy of 0.142 for bacteria and 0.781 for microspheres with the method of enhanced depth of field (EDF method). Moreover, during long-term bacterial tracking and detection, target bacteria at multiple z-axis focal depth positions can be recorded by the aid of microspheres as a positioning aid for 3D reconstruction, and the focal depth positions can be repositioned within 3–10 h. The structural similarity (SSIM) of microscopic image structure differences at the same focal depth fluctuates between 0.960 and 0.975 at different times, and the root-mean-square error (RMSE) fluctuates between 8 and 12, which indicates that the method also has good relocation accuracy. Thus, this method provides the basis for rapid, high-throughput, and long-term analysis of microscopic changes (e.g., morphology, size) of bacteria detection under the addition of antibiotics with different concentrations based on microfluidic channels in the future.

## 1. Introduction

Compared with the addition of minimal inhibitory concentrations (MIC) or lower concentrations of antibiotics close to MIC, highdose levels of antibiotics will produce inhibitory and killing effects on bacteria for a short time. However, this will easily lead to the emergence of drug-resistant bacteria. Antibiotics lower than or close to the MIC level also have a bacteriostatic effect in the short term, but the bacteria will continue to grow over a long-term period, and the effect should be reevaluated. Therefore, a long-term dose–time comprehensive effect is needed [1]. Common and emerging antibiotic susceptibility testing methods were described in recent reviews [2,3]. To speed up the detection, many studies have developed detection methods based on flow cytometry, DNA, Raman spectroscopy, polymerase chain reaction, etc. [4,5,6,7]. However, these methods also significantly increase the cost of analysis due to the expensive instruments. With the development of microfluidic devices, detection at the micrometer scale combined with microfluidic chips can not only speed up the detection time, but also be suitable for high-throughput and portable detections [8,9]. Therefore, antibiotic susceptibility testing for changes in cell growth has great commercial potential at present [10].

Time-resolved imaging can be very useful to explore the dynamics of bacterial processes, such as morphological changes induced by antibiotic overuse. Some bacteria grow into filamentous forms accompanied by other changes in area and morphology in the antibiotic environment. Thus, the study of bacterial morphological changes in response to changing environments is invaluable for the chemical and genetic profiling of drugs, or to explore heterogeneity and the role of cell shape in pathogenesis. High-throughput microscopy for studying the morphology of bacteria was recently described for this purpose [11].

By applying a microfluidic chip and a time-lapse imaging technique through a micro-scope, the high-throughput growth information of bacteria under the action of antibiotics can be quickly obtained [12,13,14,15,16,17]. Different microscopes can be used to obtain microscopic images with different characteristics. For example, high-quality fluorescence images can be obtained by using a fluorescence microscope [18]. However, due to the cost of expensive instruments and dyes, more detection expenditure will also be incurred. Moreover, since the moving bacterial fluid is not conducive to be observed in the microchannel, it is beneficial to use agarose to fix the bacteria in the microchannel culture to track the growth state of the bacteria [19,20]. Thus, the height of the culture channel is essential for long-term observation. If the channel height is too thin, there will not be a sufficient number of bacteria for detection. If the channel height is too thick, there will be multiple layers of bacteria in the focal depth of the microscope, which will also affect the uniform diffusion of antibiotics [19,20,21]. Therefore, the depth of most microchannels is selected between tens to hundreds of microns, and in most cases, multiple layers of bacteria will exist in these microchannels; that means that at a certain focal depth of the microscope, there will be a clearer picture and a blurrier picture of bacteria simultaneously, which will increase the difficulty of image processing and information extraction. Meanwhile, since the long-term time-lapse images are only focused at a certain focal depth, information from other layers in the microchannel will be lost, and the influence of bacteria in other layers will continue to expand as the bacteria grow. In addition, due to the slow growth and change of bacteria, rapid antibiotic analysis generally requires a time period of 3–10 hours, and the bacteriostatic effect is mainly reflected by changes in morphological characteristics, such as length and area [22]. However, under long-term observation over 10 hours, when tracking the growth state of bacteria, the focus range cannot be quickly fixed to find the target field of view. Although expensive equipment, such as Nikon’s PFS3 system (Perfect Focus System3, PFS3, Shanghai, China) and Olympus’s IX-ZDC (Z Drift Compensation, ZDC, Tokyo, Japan) system, can help us perform precise positioning in the z-axis direction, these instruments all require additional hardware systems, which will increase the extra high cost of detection. 

Meanwhile, though the traditional machine vision methods can be used to extract bacterial feature information from single-cell microscopic images, these methods are based on overlay features of images, and there are many different focal states of bacteria in the image at a certain focal depth under the 3D microchannel, which also have differences in morphology. Therefore, it is difficult for the image to be recognized by traditional machine vision methods for bacteria recognition with variable 3D features. For example, when the template matching method is applied for target detection, only one static template can be adopted for target recognition, and the recognition effect is single. The recognition rate will be greatly reduced when the target bacteria are distributed at different angles. Due to the change in focus state, the images of all bacterial morphologies cannot be collected as templates and applied as templates for matching targets.

To solve the problem of multiple layers of bacteria in the microchannel, we can effectively expand it into three-dimensional space. For example, the three-dimensional information of the object is reconstructed through multi-layer scanning [23], and the information of each layer in the microchannel is obtained by scanning at each focal depth in the microchannel, and a certain amount of microspheres are mixed as a positioning reference to record the depth position information [24]. To this end, it is necessary to distinguish and identify bacteria and microspheres accurately, since bacteria and microspheres are extremely small targets with few features under a large field of view, and are difficult to detect through conventional observation equipment. Due to the advantage of deep learning, it is quite feasible to analyze microscopic images via the deep learning method, and to identify and locate bacteria and microspheres [25].

Often, cells exhibit behaviors as a group that do not manifest at the single-cell level [26]. The area of interest should be selected according to the research object. However, due to the limitation of the field of view, it is difficult for a field of bacteria observation, which has a variety of different flora at the same time. Thus, under the condition of control variables, the multi-layer field of view can provide more samples. Single-layer bacteria are difficult to cultivate for a long period (lack of nutrients, limited space for bacterial growth, etc.). In addition, the tilt of the sample will cause the bacteria to focus unevenly on the focal plane in a short period, and it will also cause the image to be out of focus over a long period [27]. However, these problems can be solved by calibrating to the previous focal plane through the aid of the microspheres. Moreover, microspheres at different focusing states can be identified by the YOLO algorithm to provide marked positions as an auxiliary localization annotation.

**Figure 1:**
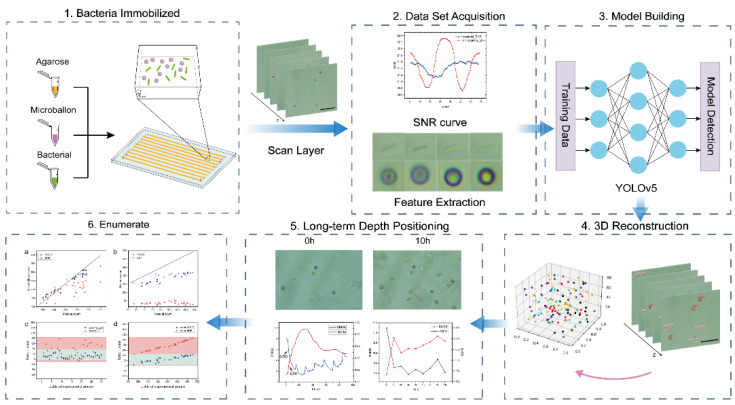
Flow chart of long-term tracking and detection of Escherichia coli at different microchannel depths.

In this paper, an ordinary optical biological microscope was used for observation, and a certain amount of microspheres were mixed to assist in locating bacteria at any focal depth, which was convenient for tracking the growth state of bacteria over a long time. A microfluidic chip with ten parallel channels was used as a bacterial culture chamber in this system, and an ordinary optical microscope and a 10-megapixel CCD camera were used for sample acquisition. Then, a deep learning model and a self-designed processing algorithm were adopted to track the growth state of bacteria at any depth for a long time. Importantly, the high-throughput time-resolved imaging methodology is quite timesaving and requires only off-the-shelf hardware and microscope software utilities. As shown in Figure 1, bacteria were cultured in a microfluidic chip (detailed process is described in SI), and a fixed field of view with the moderate bacterial density was selected (x and y directions were not moved later on). Then, by moving the z-axis, the focal plane was slowly moved from the top of the microchannel to the bottom of the microchannel, and the process was recorded by video. Second, all video frames were taken out to analyze the signal-to-noise ratio curve of bacteria and microspheres in each frame, and the bacteria and microspheres in the focused state were marked. Third, the YOLO algorithm, a deep learning model [28] was applied to train the labeled bacteria and microspheres to obtain a detection model that can identify bacteria and microspheres. The samples were recorded in the same way as in the first step, and each frame in the video was detected by the model trained in the third step. Among them, there were multiple frames recording the same focal plane. Since multiple frames were regarded as the same focal plane in the recorded video, these frames need to be deduplicated, and the coordinate information of bacteria and microspheres in the deduplicated frames was reconstructed in three-dimensional space, where the z-axis represents the different focal planes. Finally, according to the information of the 3D reconstruction in the z-axis, we can select multiple focal planes according to the requirements (such as a colony with a moderate density of bacteria, etc.) and relocate to the same focal plane in the long-term sampling observation. Thus, the parameters, such as the number of bacteria in the focal plane, could be tracked and detected. Compared with electron microscopes and fluorescence microscopes, this image sensor combined with deep learning tools does not require expensive instruments, such as three-dimensional micro-operation tables. There is also no need to add external hardware to compensate for the z-axis drift or the condition of being out of focus. Thus, bacterial morphology analysis can be performed within a 10 h period using an ordinary optical microscope and long-term video detection, providing a basis for rapid, high-throughput, and long-term bacterial–antibiotic detection based on microfluidic channels in the future.

## 2. Method

### 2.1. Video Recording

In the experiment, the bacterial solution, microspheres, and agarose were mixed evenly at 37 °C by a magnetic stirrer, and the mixed solution was injected into the microfluidic chip by a micropump, and then placed at room temperature for 10 min to solidify the mixed solution in the microchannel (Figure S1 in the Supplementary Materials). A biological microscope was used to scan and video record the layers within the microchannel (Movie S1). The experimental image sensor platform and procedure, the preparation steps of *E. coli* samples, and the design parameters of the microfluidic chip are described in detail in the Supplementary Materials.

### 2.2. Model Design

**Figure 2:**
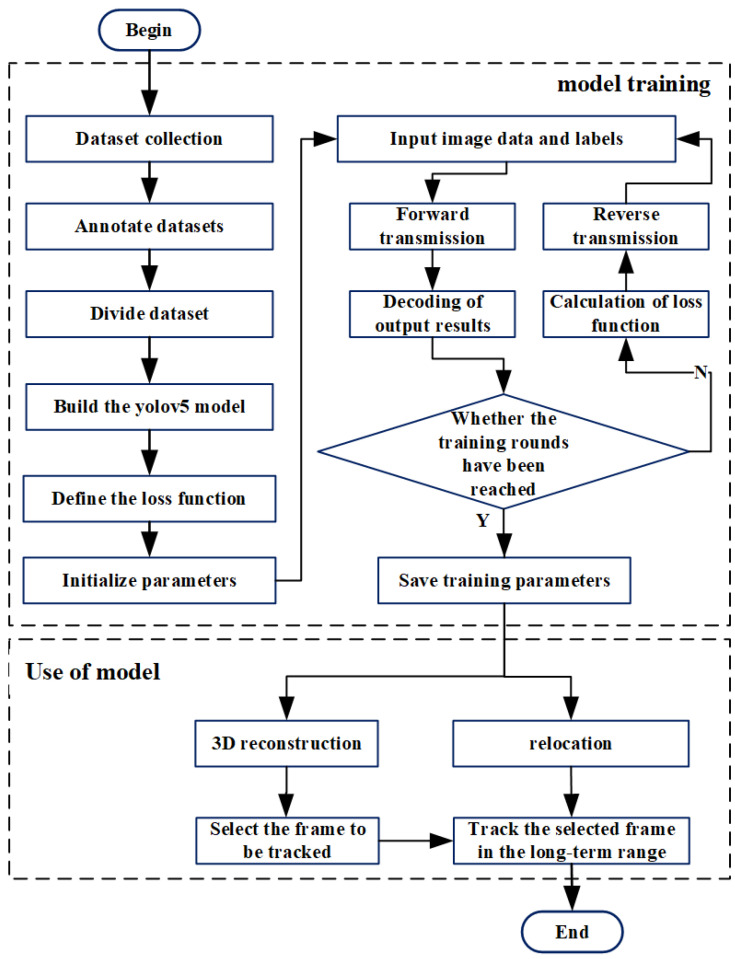
Flow chart of model training.

The target detection method used in this paper is the YOLOv5 model, which is based on the deep learning algorithm. As a mainstream target detection algorithm, it is characterized by high precision and high speed, and can also be used to detect small targets [28]. This system involved an Intel 11700 kf CPU, 32 GB of RAM, and Nvidia GeForce 3060 GPUs. In the experiments, the video samples were taken out frame by frame to obtain a picture set, and then 400 sample images with a resolution of 4912 × 3684 were randomly selected from the picture set as the dataset. Next, all dataset images were adaptively scaled to 1280 × 1280 to fit the set standard input size of the YOLO model. The value of BATCH_SIZE was set to 8, and the model was trained for 300 rounds. During the model training process, image scaling, color space adjustment, and Mosaic-8 enhancement methods were used to enhance the image data and increase the number of training samples. The dataset was divided into a training set and a validation set, and the ratio of the training set to the validation set was 0.8 to 0.2. There were 320 frames of images in the training set, including 1952 bacterial samples and 2701 microsphere samples. A total of 80 frames of images were selected as the validation set, including 506 bacteria and 729 microsphere samples. The three indicators of precision, recall, and F1 were applied to evaluate the model under different background noises [29]. As shown in the flow chart of training and use of the model in Figure 2, the process of model building is mainly divided into the training process and the application process.

### 2.3. Comparison of 2D and 3D Detection Methods

In the time-lapse image of the bacterial growth state over a long period, when the focal depth remains unchanged, only the time-lapse image of the bacteria at a certain microchannel depth can be obtained, and the entire z-axis range in the microchannel cannot be displayed. The quality of these time-lapse images will be directly affected by the number of bacteria and the amount of information at the focal depth. To obtain information on images at all focal depths over the entire z-axis range, two approaches can be used. The first approach is to superimpose the image information of each z-axis section to obtain a stacked feature image, and the other approach is to scan the image at each focal depth with the change of the z-axis to obtain images and videos at all focal depths. Although the stacking method can reduce the three-dimensional to two-dimensional and lower the processing difficulty, all the information of each layer must first be merged, which will lead to a decrease in processing efficiency. Secondly, to better integrate the image information of each layer, the information, such as bacterial contour features, will be severely reduced, and the interference of noise will also be increased. Furthermore, when the bacterial concentration is higher, multiple bacterial stacking often occurs. Therefore, by using the z-axis scanning method, not only can the morphological characteristics of bacteria and microspheres be preserved, but the density of bacteria and microspheres can be greatly reduced, which is conducive to the tracking and detection of bacteria.

### 2.4. 3D Reconstruction and Tracking Algorithm

Since the method of frame-by-frame detection was applied to detect the video, the same object will appear in different image frames with different forms, which leads to the problem of a single object being repeatedly detected multiple times. To better select the focal depth of the target to be tracked, the same bacteria repeated in the upper and lower frames needed to be removed, and the location of bacteria in each focal depth layer after deduplication was reconstructed three-dimensionally so that the location of bacteria in each focal depth layer could be displayed intuitively; this was convenient for the subsequent selection of suitable focal depth layers for tracking. Confidence is a parameter in the YOLO model that represents the degree of confidence in identifying objects [30,31]. Since the confidence of the same bacteria changed monotonically as the focal depth approached, the rest of the same detected bacteria in the non-focused state could be removed through the change of confidence. Finally, the unique coordinate point of each target after deduplication could be obtained. For each target bacteria layer, unique coordinate points were applied for reconstruction in three-dimensional space, so the number of bacteria on each focal depth layer could be visually displayed, and an appropriate layer or layers could be selected for tracking. The specific implementation process of deduplication and relocation can be seen in Supporting Information.

Simultaneously, to track the time-lapse images under one or more focal depth layers, microspheres were used as positioning aids to record the position information under the focal depth layers. Since the position of microspheres was not changing with time after being fixed with agarose, the position, number, and confidential information of the microspheres at different focal depth positions at each sampling moment could be recorded, and the time-lapse images of bacterial growth at the same focal depth could be obtained by searching for images with highly similar microsphere information at each sampling moment.

## 3. Results

### 3.1. Performance Comparison with Other Models

To compare the YOLOv5 algorithm with the YOLOv3 and YOLOv4 algorithms, the same image input was used, and the training rounds were uniformly set to 300 rounds. As shown in Table 1, when comparing the average precision [32] of the two objects side by side, YOLOv5 had the satisfactory average detection precision, and the detection precision of YOLOv5 on microspheres was significantly higher than that of YOLOv3 and YOLOv4, which can provide more accurate microsphere location information for subsequent relocation.

### 3.2. Performance Comparison of 2D and 3D Detection Methods

**Figure 3:**
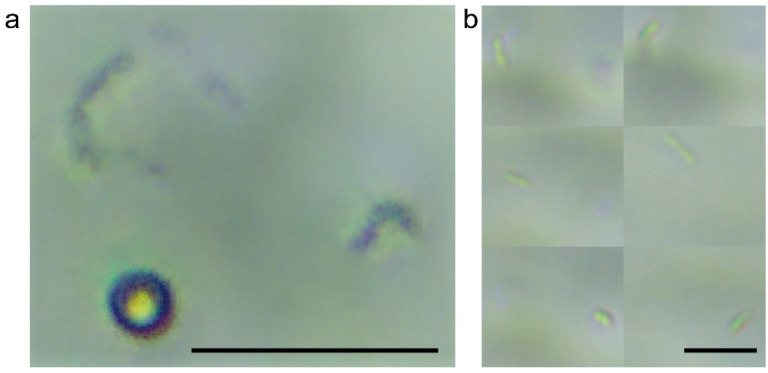
Comparison of stacking method (2D) and layer-by-layer scanning method (3D): (**a**) Enhanced depth of field mode (2D); (**b**) layer-by-layer scanning mode (3D). The scale bars represent 10 μm.

As shown in Figure 3a, the bacterial features of each scanning layer were stacked on one image through the method of enhanced depth of field (EDF method). Figure 3b is an image of the bacterial layer using the layer-by-layer scanning method. It shows that bacterial stacking occurs with the EDF method, and the bacterial characteristics are severely reduced. Compared with the method of layer-by-layer scanning, the EDF data of bacterial liquid under the target field of view was not easy to detect accurately. Both these methods were often used to recognize the target bacteria layer. However, the accuracy of the layer-by-layer scanning method was much higher than that of the 2D stacking method compared with the method of manual counting. The comparison result of the approximate value range showed that the 2D stacking method was mainly based on features of enhanced depth of field, which could be seriously lost with different occasions. In terms of bacterial counting accuracy (Equation (4)), the accuracy of the 3D method was more than 75% higher than that of the 2D method, and the accuracy of the 3D method was more than 10% higher than that of the 2D method in terms of microsphere counting accuracy.

### 3.3. Denoising Performance Evaluation

**Figure 4:**
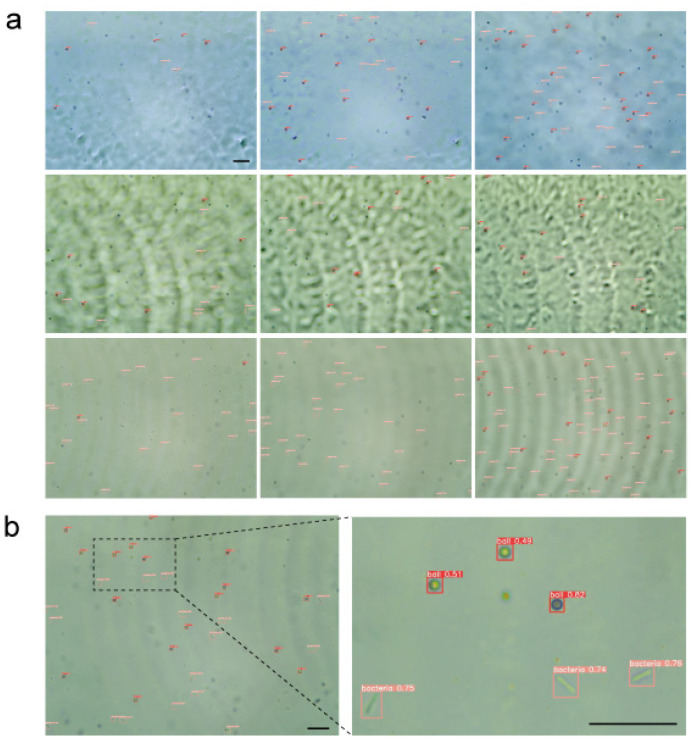
Recognition effect: (**a**) The recognition effect under different backgrounds and in low, medium, and high concentrations of bacteria liquid, respectively; **(b**) Enlarged picture of recognition effect under normal background. The scale bars represent 20 μm.

**Figure 5:**
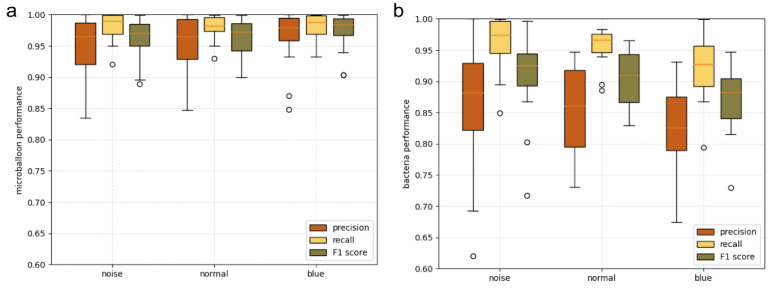
Performance description with box diagram as precision (red), recall (yellow), and F1 score (green) obtained with the blue background stained with trypan blue (N = 20), loud noise background (N = 20), and normal background (N = 20) datasets. (**a**) microballoon recognition performance; (**b**) bacteria recognition performance.

In the process of long-term relocation and analysis of bacterial liquid, the analysis of different background noises is particularly important. After 100 rounds of training, the overall evaluation index of the model no longer decreased, and the model automatically terminated after 206 rounds of training. Therefore, the model parameters of the 106th round with the best training results in the 206 rounds of training were taken as the final model, and the detection result is shown in Figure 4b. Different color backgrounds and different sources of noise interference were adopted to analyze the background noise. As shown in Figure 4a, the detection was carried out mainly under the blue background (stained with trypan blue), the large noise background, and the normal background. As shown in Figure 5, Equations (1)–(3) were adopted to calculate the three indicators of precision, recall, and F1 for verification at the sampling time of the first hour. Under three different backgrounds, the average values of precision, recall, and F1 of microspheres were above 0.95, and the data were more concentrated than those of bacteria, which indicates that the recognition effect of microspheres is ideal and stable under different backgrounds. Compared with microspheres, the values of the precision of bacteria were relatively scattered, but the average values of precision were also above 0.82, which indicated that the recognition accuracy of bacteria was also relatively high. The reason for the decrease in precision is that bacteria have more morphological changes than microspheres, which leads to more bacteria not being detected. At the same time, the average values of bacterial recall were more than 0.92 and more concentrated than precision, which indicates that the detected target was rarely misjudged and the detection effect was stable. Therefore, both bacteria and microspheres have satisfactory recognition effects under different backgrounds.

(1) $$Precision=\frac{TP}{TP+FP}$$

(2) $$Recall=\frac{TP}{TP+FN}$$

(3) $$F1=\frac{2\times Precision\times Recall}{Precision+Recall}$$

In the above formula, *TP* is the number of bacteria or microspheres that are correctly identified as bacteria or microspheres, *FP* is the number of bacteria or microspheres that are incorrectly identified as bacteria or microspheres, and *FN* is the number of bacteria or microspheres that are not identified.

### 3.4. Counting Performance Evaluation

**Figure 6:**
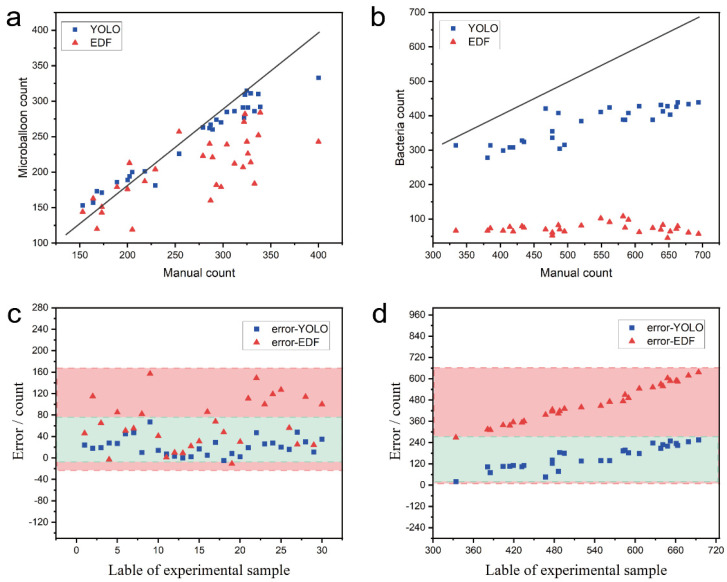
Comparison of microsphere and bacterial counting results between stacking and layer-by-layer scanning: (**a**) microballoon; (**b**) bacteria; and counting deviation comparison for microsphere and bacteria: (**c**) microsphere; (**d**) bacteria.

To reflect the concentration of bacteria and microspheres, it is necessary to accurately count the bacteria and microspheres in the microchannel. Both 2D and 3D methods were adopted to count microspheres and bacteria of each layer. The microsphere counting results of 30 randomly selected sample groups by the three-dimensional scanning method (YOLO) and the method of enhanced depth of field are shown in Figure 6a. The counting result of the three-dimensional method was closer to the actual manual counting result, which indicates that the three-dimensional method is better than the method of enhanced depth of field. The error and deviation diagram of the two methods on the microsphere counting results are shown in Figure 6c. The blue dotted line represents the maximum and minimum error values of microsphere counting under 3D mode, and the red dotted line represents 2D mode. The counting width of the red line is 168, and it is much larger than the width of the blue line with the size of 72, which indicates the error of the method of enhanced depth of field is significantly higher than that of the three-dimensional scanning method. Similarly, the counting results of bacteria with these two methods are shown in Figure 6b, and the error and deviation of the counting results are shown in Figure 6d, which also shows that the 3D scanning method is better than the method of enhanced depth of field. By comparing the counting results of 30 groups of bacteria, the maximum counting errors (Equation (5)) in the 3D method and the 2D method were 255 and 637, respectively, the mean errors were 158.17 and 460.17, and the standard deviations were 64.93 and 105.37, respectively, which shows that the 3D mode is significantly better than the 2D mode. However, the count error is larger than that of microspheres, because microspheres are easier to identify than bacteria. Moreover, with the increase in the number of bacteria, the deviation of the two counting methods gradually increases, and the error on the method of enhanced depth of field is greater. It is pointed out by some researchers that under a low concentration of bacterial solution, the long-term growth change of bacteria under the action of antibiotics is linearly consistent with the concentration of antibiotics [33]; therefore, it is particularly significant for the long-term accurate positioning and tracking of bacteria under low concentration bacterial liquid distribution. As the result shows, in the 3D layer-by-layer scanning method, the bacterial counting accuracy and microsphere counting accuracy were 0.592 and 0.129 higher than those in the 2D method of enhanced depth of field, respectively, which shows the concentration of bacteria and microspheres can be better recognized by the three-dimensional counting method.

(4) $$counting\;accuracy=\frac{Number\;of\;model\;counts}{The\;real\;number}$$

(5) counting error=The real number−Number of model counts

In the above formula, *Number of model counts* is the number of bacteria/microspheres calculated by the model, *The real number* represents the number of bacteria/microspheres by manual counting.

### 3.5. 3D Reconstruction and Bacterial Tracking

**Figure 7:**
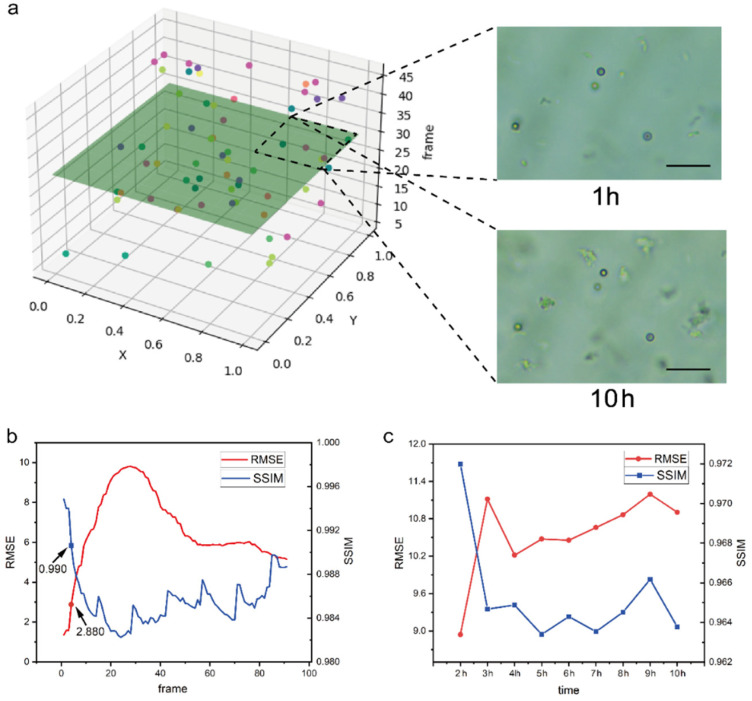
3D reconstruction and tracking of bacteria over a 10 h long term: (**a**) Reconstruction of 3D coordinate and selection of target frame; (**b**) Changes in RMSE and SSIM values between the relocated target frame and the rest of the frames under repeated scanning; (**c**) Fluctuation of RMSE and SSIM values in target frame under long-term tracking. The scale bars represent 20 μm.

The main problem with the microsphere-assisted three-dimensional positioning method is that the bacteria and microspheres in each frame can be identified by the YOLO model, but the same bacteria or microspheres will appear in multiple frames with different focus states, so the images need to be deduplicated and the detailed implementation process is described in Supporting Information. At the same time, since there are too many frames in each video sample, the tracking efficiency will be greatly reduced, and repeated target frames will be easily selected if all frames are tracked. Therefore, it is necessary to facilitate the relocation of the target bacteria so that the same focal plane is observed in the tracking observation. In this paper, a three-dimensional reconstruction of the bacteria coordinate data after deduplication was carried out, the position coordinates of bacteria in the microscopic image were taken as the x-axis and y-axis, the number of frames in the video sample was taken as the z-axis, and the same focus depth position was represented by the same color. As shown in Figure 7a, the focal depth layer with appropriate number bacteria was selected as the target layer, and the coordinates of the microspheres of the focal depth layer were marked. After video scanning sampling was performed at the next detection time, the image frame with the same focal depth layer was found again through the positioning information of the microspheres (the detailed implementation process is described in Section S2.2 of S1). Finally, the long-term tracking and detection of bacteria at the same coordinate were completed. To verify the accuracy of the relocation, the same sample was scanned during a 10 h period at 1 h and 10 h, and the difference between the images was quantified by structural similarity (SSIM) and root-mean-square error (RMSE) [34,35]. As shown in Figure 7b, to scan the same sample twice in a row, a frame in the first scan was randomly selected, and then the RMSE and SSIM values of this frame and all frames in the second scan were calculated. If the RMSE and SSIM values of relocation are close to the maximum value, it represents that the structure feature of the relocated frame is highly similar to the structure of the selected frame. As shown in Figure 7c, during the long-term culture of bacteria, the same focal depth could be relocated at each sampling time point. Compared with the changes in the RMSE and SSIM values of the target frame selected in the first hour, the data of the first 3 hours changed obviously. However, the change of data fluctuation decreased after 3 hours. This situation could be attributed to the fact that in the first 3 hours, the growth rate of bacteria was fastest, which resulted in the largest change in the structure of the microscopic image. Thus, although the same point of focus depth was relocated, there were changes in the image structure due to the growth increase in bacteria, and the coordinates of the microspheres are necessary.

## 4. Discussion

In this study, a method for tracking bacterial growth states at different depths of microchannels was provided. At each sampling time, the YOLO model was used to identify Escherichia coli under different focusing states at a certain focal depth. Simultaneously, microspheres were supplemented as a positioning aid, and the invariable information, such as the position and state of microspheres, was used to locate the depth position of the previous sampling time to track the changes of growth state of the target bacteria. The extraction of image features of bacterial changes over a long period of time also provides conditions for antibiotic susceptibility testing.

We demonstrated the rationality of applying a three-dimensional scanning mode to solve the problem of multi-layer bacteria in microchannels and compared the recognition effect of bacteria in 2D mode with that in 3D mode. Due to the stacking of features, there is a large error in the bacterial count results in 2D mode. In addition, we also explained the reason why 3D mode is better than 2D mode. In the meantime, microspheres were adopted as a positioning aid for long-term tracking of bacterial growth. The time for antibiotic susceptibility testing can be greatly accelerated by observing the doubling of a single bacteria directly, which is of great significance to obtain time-lapse images of single cells for this purpose [18]. The growth status of bacteria can be tracked, and time-lapse images of single cells can be obtained by using agarose microchannels [18,19,20]. However, due to the limited depth of field of the microscope, each layer of bacteria in the microchannel cannot be observed at the same time, and only a fixed focal depth can be selected to track and observe the bacteria in this layer. Meanwhile, the bacteria that can be observed will remain in different focusing states under a fixed focal depth. Although the depth of the microchannel can be reduced to only a single layer of bacteria in each microchannel, it will greatly reduce the actual number of bacteria for testing and shorten the culture life of bacteria, resulting in the inability to obtain correct identification results, which is not conducive to the long-term morphological tracking and analysis of bacteria under the action of antibiotics. Moreover, studies have shown that the survival of cells with grossly altered morphology after antibiotic action not only depends on their ability to withstand lysis, but also on their successful post-antibiotic recovery back to normally growing cells. This requires recording the precise position of the target bacteria in three dimensions and tracking their long-term morphology.

To obtain a sufficient number of bacteria for tracking their growth status, considering that the focal depth position at each sampling moment can be recorded by applying the microsphere as a positioning aid, it is possible to track the microscopic image of bacteria at multiple focal depths of microchannel depths of the same sample simultaneously according to the microsphere-assisted positioning, which provides more effective data for the morphological analysis of the target bacteria under the effect of antibiotic susceptibility testing. In this study, the deep learning algorithm based on YOLOv5 was used to detect the growth images of multiple target bacteria in microchannels with different depths. The identification of multi-layer bacteria and microspheres in microchannels can effectively solve the problem of multi-layer bacteria identification, and has high accuracy with different interference backgrounds, and in high, medium, and low concentrations of bacteria liquid. Therefore, the deep learning algorithm based on YOLOv5 is especially suitable for the recognition of bacteria with morphological changes in the z-axis direction. At the same time, the auxiliary aid of the microspheres in the three-dimensional coordinate system was applied to track the change in different growth states of the same bacteria and analyze the antibacterial effect in the antibiotic susceptibility test. Since the morphology of the microspheres remains stable, it is quite suitable for a 10 h long-term positioning observation. Compared with the method of enhanced depth of field, the counting accuracy of bacteria and microspheres was significantly improved, which could reflect the fitting relationship between the concentration of bacteria and the concentration of antibiotics in the later stage. Finally, based on the invariable characteristics of the microspheres, the bacteria in the region of interest were relocated over a 10 h long period by using the recognition data of the microspheres, which was convenient to track and observe the changes in the morphology and area of the region of interest under antibiotics. In follow-up studies, this method is worth being applied in the development of automated analysis platforms to obtain the minimum inhibitor concentration of antibiotics quickly and to explore the application of rapid antibiotic susceptibility testing.

## 5. Conclusions

The YOLO detection method applied in this paper can accurately identify Escherichia coli and microspheres in different focusing states at a certain focal depth. The recognition accuracy and counting accuracy of bacteria were 0.734 and 0.714, respectively, and the two recognition rates of microspheres were 0.910 and 0.927, respectively, which were much higher than the counting accuracy of 0.142 for bacteria and 0.781 for microspheres with the method of enhanced depth of field. The detection method applied in this paper is focused on the area and other features of bacteria while avoiding the influence of background, impurities, long period of time, and other interfering factors in a large field of view. Secondly, the growth state of bacteria can be better tracked and observed throughout the whole experiment process, which facilitates the extraction of image features of bacteria, such as area changes and morphological changes. Furthermore, when capturing the information on bacterial growth and changes over a long period, it is difficult to relocate to the original focal depth position at the next observation moment after the focal point changes. Therefore, the microsphere was applied as an auxiliary aid to locate the same location in multiple scan samples, which realized the same focal position to be rapidly relocated within 3–10 h during the growth change of the bacteria. The value of the microscopic image structure difference (SSIM) at the same focal depth fluctuated between 0.960 and 0.975 in the whole experiment process, and the value of the root-mean-square error (RMSE) fluctuated between 8 and 12. Finally, with the assistance of microspheres, the same position can be located at multiple sampling moments, so multiple bacterial samples at different microchannel depths can be recorded to observe more samples of bacterial growth status at each moment. This work provides an image sensor platform with deep learning tools for future studies, including the long-term detection of bacterial morphology, activity analysis, and structural changes in cellular components etc., in bacteria under the action of a variety of trace antibiotics.

## Figures and Tables

**Table 1:** The results of the comparison of YOLOv3, YOLOv4, and YOLOv5 detection algorithms.

Model	Precision/Ball	Precision/Bacterial	mAp@0.5
YOLOv3	0.820	0.686	0.754
YOLOv4	0.849	0.729	0.789
YOLOv5	0.91	0.734	0.822

## Data Availability

Data available on request from the authors.

## Supplementary Materials

The following supporting information can be downloaded at: https://www.mdpi.com/article/10.3390/s22197454/s1. Figure S1. Sample preparation: (a) Schematic diagram of the preparation process; (b) Microscopic images of microfluidic chips and samples. The scale bars represent 20 μm. Figure S2. The focused state of bacteria and microspheres.
