# Supplementary material for: YOLO Algorithm for Long-Term Tracking and Detection of Escherichia Coli at Different Depths of Microchannels Based on Microsphere Positioning Assistance
Source: Sensors (Basel). 2022 Sep 30;22(19):7454. doi: 10.3390/s22197454 (PMC9572565; doi:10.3390/s22197454)
Supplement: Supplementary file 1 [file sensors-22-07454-s001.zip › sensors-1929091-supplementary/supplementary/supplementary.pdf]

Supporting Information

YOLO algorithm for long-term tracking and detection of Escherichia coli at different depths of microchannels based on microsphere positioning assistance

Content

Supporting Information **1**

S1. Experimental section **2**

S1.1 Experiment.. 2

S1.1.1 E.Coli Culture.. 2

S1.1.1 Sample preparation.. 2

S1.2 Equipment.. 3

S1.3 Design of the microfluidic chip.. 3

S2. Data Analysis **4**

S2.1 Annotation of the dataset.. 4

S2.2 Duplicates removal and relocation.. 5

S1. Experimental section

S1.1 Experiment

S1.1.1 E.Coli Culture

The E. coli adopted in the experiment was from DH5α competent cells of Shanghai Sangon Bioengineering Co., Ltd. The E. coli was inoculated into the autoclaved LB agar medium by the streak method and then was placed in a 37°C incubator for 36 hours. Then, an independent colony with good growth conditions was selected, and samples were moved to a sterilized liquid medium for further cultivation. Finally, the bacterial liquid with the luminosity values of 0.128, 0.278, 0.308, 0.412, 0.463, and 0.626 at the wavelength of 600 nm was selected as the standard bacterial liquid sample.

S1.1.1 Sample preparation

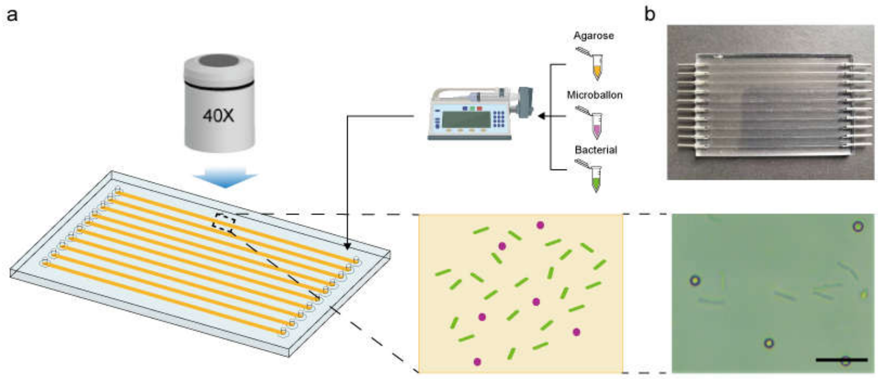

Figure S1 Sample preparation：（a）Schematic diagram of the preparation process;（b）Microscopic images of microfluidic chips and samples. The scale bars represent 20 μm.

As shown in Figure S1, 2ml standard bacterial solution, 2ml 2μm-diameter microsphere solution that was diluted by 32 times (Shanghai Yiyuan Biotechnology Co., Ltd.), and 1% low melting point agarose (Shanghai Keyizhe Electromechanical Engineering Co., Ltd.) were aspirated and then injected into a 10ml beaker. A constant temperature magnetic drive stirrer was used to fully mix the solution at 37°C, and a micropump was used to inject the mixed solution into the microfluidic chip. The chip was left at room temperature for 1 min to solidify the sample in the channel. 6 different

concentrations of Escherichia coli bacteria were mixed with microspheres and agarose, respectively, and were injected into the microchannel to obtain 30 microchannel samples. The microchannels were video-recorded by using a biological microscope equipped with a 40× objective lens and a 20× electron microscope attached to a 10-megapixel CCD camera and 10 different fields of view were randomly selected for each microchannel. During the video recording, the focus of the biological microscope was gradually adjusted from the top of the microchannel to the bottom of the microchannel, and a video sample was obtained for each field of view. Each image frame in the video sample corresponded to images with different depths of the microchannel. Since each of the 30 microchannel samples had 10 fields of view, 300 video samples were obtained.

S1.2 Equipment

The microfluidic chip was customized by Beijing Lishi Micronano Technology Co., Ltd. The CM2000S infinity plan achromatic biological microscope was purchased from Nanjing Nanpai Technology Co., Ltd. The dual-channel syringe pump was purchased from Zibo Guanjie Electronic Technology Co., Ltd. The 10-megapixel CCD camera was purchased from Henan Baopin Technology Co., Ltd. The SX-500 high temperature and autoclave sterilizer was purchased from TOMY. The ZHJH-C1106C ultra-clean bench was purchased from Zhengzhou Nanbei Instrument Equipment Co., Ltd. The MCO-15AC carbon dioxide cell incubator was purchased from Sanyo. The BSA124S-CW precision balance was purchased from Sartorius. The UV-visible Spectrophotometer UV-2600 was purchased from Shimadzu Corporation. The micropipette was purchased from Eppendorf. Escherichia coli strains were purchased from Sangon Bioengineering (Shanghai) Co., Ltd. All bacteria-related reagents were sterilized at high temperature and high pressure.

S1.3 Design of the microfluidic chip

Due to the good temperature resistance and light transmittance of the

polycarbonate material (PC) chip, and taking the need for a good light transmittance to observe the inside of the microchannel by using microscope, the PC material was selected. It also has the advantage of easy observation of the agarose melting process at a certain temperature. In addition, considering the size of the field of view under the 40x objective lens, and the minimum limit of the runner width of the PC material chip was 0.5mm, the runner width was designed to be 0.5mm. Since each focusing layer within the depth of the runner needs to be photographed, the deeper the runner depth, the more focusing layers. Therefore, the depth of the runner was designed to be 0.1mm, because the minimum limit of the runner depth of the PC material chip was 0.1mm. At the same time, different areas of the flow channel should be randomly sampled, so the length of the flow channel should also meet the requirements of sufficient number of sampling points. Thus, the effective length of the flow channel was designed to be 60mm, and the thickness of the PC substrate was about 4mm. Because the focus range of the microscope decreases with the increase of magnification, a 0.2 mm thick PC film bond was adopted to enable the region within the entire microchannel depth to be observed. The chip consists of ten linearly independent channels with the same length, width as well as depth.

S2. Data Analysis

S2.1Annotation of the dataset

Usually, the manual labeling method is more accurate than the automatic labeling method. To make the labeling more consistent, the dataset can be labeled by two labelers and the common part is selected as the labeling results[1]. To carry out effective labeling, the standard of labeling was developed by calculating the change of the signal-to-noise ratio of the target object and setting the threshold of the change jointly by multiple researchers. As shown in Figure S2, to extract typical local images which contained bacteria and microspheres with different focusing degrees, a straight line was cut through the center of the microspheres, then formula 1 was adopted to calculate the

signal-to-noise ratio of each point on the line and the maximum signal-to-noise ratio range on the cross-section. Finally, the state with a range threshold greater than 1 was selected as the focus state of the microsphere. By using the same method as above, a state with a range threshold greater than 0.15 was selected as the focus state of bacteria.

$$SNR = 10 \cdot \log_{10} \frac{g_i}{stdDev} \quad (1.)$$

In the formula, SNR, gi, and stdDev represent the signal-to-noise ratio, the gray value of point i on the intercept, and the standard deviation of the background, respectively.

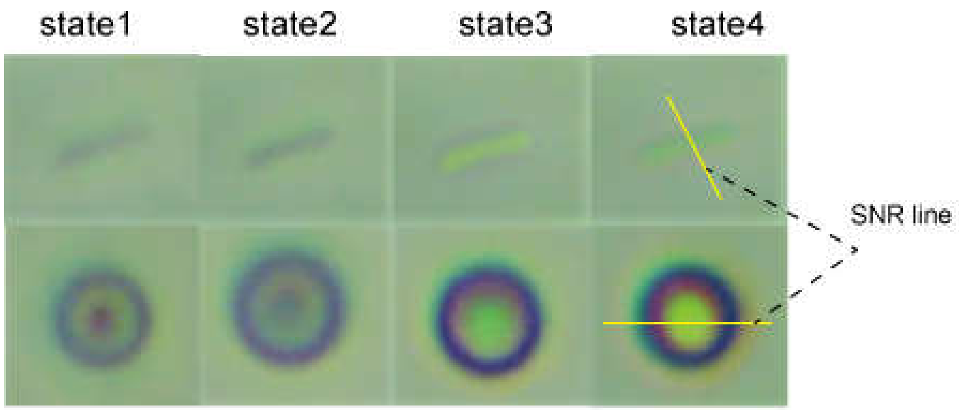

Figure S2 The focused state of bacteria and microspheres

S2.2 Duplicates removal and relocation

Although the bacteria and microspheres in each frame were detected, the counting and three-dimensional coordinate position reconstruction of bacteria and microspheres would be greatly affected if the same bacteria or microspheres were detected in adjacent frames at the same position. The bacteria and microspheres in all frames at the same position and adjacent to each other were grouped. Since different depth positions of the corresponding bacteria or microspheres could be represented by the corresponding bacteria or microspheres in each group, the actual number of bacteria or microspheres could be set as the number of groups. The depth position with the highest confidence in each group was selected as the center position of bacteria or microspheres, and the

position coordinates were used to reconstruct the three-dimensional position coordinates, which could intuitively show the distribution state of bacteria and microspheres. This method was convenient to choose a suitable depth position for bacterial tracking.

During long-term bacterial growth, bacteria would continue to divide, which resulted in the agglomeration of bacteria. Therefore, agglomerated bacteria could not be identified by the model, and the microspheres were adopted as a localization aid to track bacteria at the same depth location over a long-term period. Firstly, the coordinate frame information of all the microsphere positions in the target frame was recorded. Then, in all the microspheres at every other moment, the frame with the largest overlap area at the position coordinate frame of the target frame was selected, which also represented the frame at the same depth position of the previous moment.

References

1. Sità, L.; Brondi, M.; Lagomarsino de Leon Roig, P.; Curreli, S.; Panniello, M.; Vecchia, D.; Fellin, T., A deep-learning approach for online cell identification and trace extraction in functional two-photon calcium imaging. *Nature communications* **2022,** 13, (1), 1-22.
